# Carrageenan-containing over-the-counter nasal and oral sprays inhibit SARS-CoV-2 infection of airway epithelial cultures

**DOI:** 10.1152/ajplung.00552.2020

**Published:** 2021-02-09

**Authors:** Desiree Schütz, Carina Conzelmann, Giorgio Fois, Rüdiger Groß, Tatjana Weil, Lukas Wettstein, Steffen Stenger, Alexander Zelikin, Thomas K. Hoffmann, Manfred Frick, Janis A. Müller, Jan Münch

**Affiliations:** ^1^Institute of Molecular Virology, Ulm University Medical Center, Ulm, Germany; ^2^Institute of General Physiology, Ulm University, Ulm, Germany; ^3^Institute for Microbiology and Hygiene, Ulm University Medical Center, Ulm, Germany; ^4^Department of Chemistry and iNano Interdisciplinary Nanoscience Centre, Aarhus University, Aarhus, Denmark; ^5^Department of Otorhinolaryngology, Head and Neck Surgery, Ulm University, Ulm, Germany

**Keywords:** carrageenan, sulfated polysaccharides, virucidal, virus inhibition, virus transmission

## Abstract

Pharmaceutical interventions are urgently needed to prevent severe acute respiratory syndrome coronavirus 2 (SARS-CoV-2) infection and transmission. As SARS-CoV-2 infects and spreads via the nasopharyngeal airways, we analyzed the antiviral effect of selected nasal and oral sprays on virus infection in vitro. Two nose sprays showed virucidal activity but were cytotoxic precluding further analysis in cell culture. One nasal and one mouth spray suppressed SARS-CoV-2 infection of TMPRSS2-expressing Vero E6 cells and primary differentiated human airway epithelial cultures. The antiviral activity in both sprays could be attributed to polyanionic ι- and κ-carrageenans. Thus, application of carrageenan-containing nasal and mouth sprays may reduce the risk of acquiring SARS-CoV-2 infection and may limit viral spread, warranting further clinical evaluation.

## INTRODUCTION

The coronavirus disease 2019 (COVID-19)-causing agent, severe acute respiratory syndrome coronavirus 2 (SARS-CoV-2), emerged at the end of 2019 and quickly spread within the human population around the globe (1). Manifestations range from mild common cold symptoms to severe lung injury, multiorgan dysfunctions, and eventually death, especially in the elderly or patients suffering from comorbidities (2). Measures to confine the spread of the virus include lockdown strategies, which severely affect socioeconomic structures. SARS-CoV-2 is mainly transmitted via respiratory droplets and aerosols exhaled from infected individuals and subsequent exposure of the respiratory mucosa of an uninfected individual (3–5). Agents reducing viral loads in the throat and nasal cavity or protecting mucosal tissue from initial infection may prevent infection and reduce virus spread between individuals (6, 7). Sprays applied to the nasal and oral mucosa to soothe symptoms, reduce disease duration, and increase viral clearance of respiratory infections caused by viruses such as rhino-, influenza-, or common cold coronaviruses have been approved and are available as over-the-counter medicine. Some contain decongestant compounds like xylometazoline (8), tramazoline, or oxymetazoline (9) to reduce symptoms of nasal congestion (10). This effect is supported by moisturizing or gel-forming mucoprotective substances such as dexpanthenol (9) and hydroxypropyl methylcellulose (11, 12). In addition, sulfated polysaccharides such as carrageenans are included as broad-spectrum antiviral agents (13–17).

As SARS-CoV-2 infects the nasopharyngeal airways, we here analyzed one oral and five nasal sprays (Table 1) for their virucidal and antiviral activity against SARS-CoV-2. All sprays are commercially available and do not require prescription. Two of the sprays exert direct virucidal activity at high concentrations but also elicited cytotoxic effects. Two carrageenan-containing sprays inhibited SARS-CoV-2 infection of immortalized cells and, more importantly, fully differentiated human airway epithelial cells resembling a crucial entry portal of the virus, with little to no effect on cell viability. Thus, application of these sprays may help to prevent from acquiring SARS-CoV-2 or suppress viral replication in the nasal epithelia in infected individuals, which may result in attenuated disease and reduced transmission rates. Further evaluation of antiviral nose sprays in clinical studies is warranted.

**Table 1.** Overview and composition of tested products A–F

Product	Trade Name	Active Agent	Additives
A	Viruseptin (nasal)	ι- and κ-carrageenan (1.2 and 0.4 mg/mL)	Sodium chloride
B	Viruseptin (oral)	ι-carrageenan (1.2 mg/mL)	Sodium chloride, xylitol, cherry flavor
C	Nasic (nasal)	Xylometazoline hydrochloride (0.1%), dexpanthenol (5%)	Benzalkonium chloride, monopotassium phosphate, disodium phosphate dodecahydrate
D	Rhinospray (nasal)	Tramazoline hydrochloride (1.264 mg/mL)	Sodium chloride, citric acid, benzalkonium chloride, menthol, cineol, camphor racemic, sodium hydroxide, magnesium sulfate, magnesium chloride, calcium chloride, sodium hydrogen carbonate, povidone-iodine glycerol 85%, hypromellose
E	Wick Erste Abwehr (nasal)	Hydroxypropyl methylcellulose	Succinic acid, disodium succinate, pyroglutamic acid
F	Wick Sinex Avera (nasal)	Oxymetazoline hydrochloride (0.5 mg/mL)	Sorbitol, trisodium citrate, polysorbate 80, benzyl alcohol, citric acid, benzalkonium chloride, acesulfame potassium, menthol, cineol, sodium edetate, aloe dry extract, l-carvone

## MATERIALS AND METHODS

### Reagents

Viruseptin nasal and oral sprays were obtained from Hälsa Pharma GmbH, Nasic from Klosterfrau Berlin GmbH, Rhinospray from Sanofi-Aventis, and Wick Erste Abwehr and Wick Sinex Avera from Wick Pharma, Procter & Gamble GmbH. ι- and κ-carrageenan were purchased from Sigma.

### Cell Culture

All cells were cultured in Dulbecco’s modified Eagle’s medium (DMEM, Gibco) containing 100 U/mL penicillin, 100 μg/mL streptomycin, and 2 mM l-glutamine. Vero E6 (*Cercopithecus aethiops* derived epithelial kidney) medium was supplemented with 2.5% heat-inactivated fetal calf serum (FCS), 1 mM sodium pyruvate, and 1× nonessential amino acids. Caco-2 (human epithelial colorectal adenocarcinoma) cells (kindly provided by Holger Barth, Ulm University) were supplemented with 10% FCS. TMPRSS2-expressing Vero E6 cells [kindly provided by the National Institute for Biological Standards and Control (NIBSC), No. 100978] were supplemented with 10% FCS and 1 mg/mL geneticin.

### Generation of Air*-*Liquid Interface Cultures of Human Airway Epithelial Cells

Differentiated air-liquid interface (ALI) cultures of human airway epithelial cells (HAECs) were generated from primary human basal cells isolated from airway epithelia, as recently described (18). Cells were isolated from tissue obtained from a male and a female donor in the age range of 25–50 yr. All experiments were performed with approval of the ethics committee of Medical School Hannover (Project No. 2701-2015). In short, 3.5 × 10^4^ cells were seeded onto the apical side of collagen-coated, 6.5-mm Transwell filters (Corning Costar) in 200 µL of apical and 600 µL of basolateral growth medium. After 48 h, the apical medium was replaced, and after 72–96 h, upon confluency, it was completely removed (air lifting). Then, the basolateral medium was replaced by differentiation medium, consisting of DMEM-H and LHC Basal (1:1) (Thermo Fisher) supplemented with Airway Epithelial Cell Growth Medium Supplement Pack, and was replaced every 2 days. Air lifting defined *day 0* of ALI culture, and experiments were performed at *days 25*–*28*. To avoid mucus accumulation on the apical side, cells were washed apically with PBS for 30 min every 3 days from *day 14* onward.

### Virus Strain and Virus Propagation

Viral isolate BetaCoV/France/IDF0372/2020 (No. 014 V-03890) was obtained through the European Virus Archive global. Virus was propagated by inoculation of 70% confluent Caco-2 cells in 75-cm^2^ cell culture flasks in medium containing 15 mM HEPES. Three days after inoculation, when a strong cytopathic effect (CPE) was visible, supernatants were harvested. Supernatants were centrifuged for 5 min at 1,000 *g* to remove cellular debris, aliquoted, and stored at −80°C. Infectious virus titer was determined as plaque-forming units, as previously described (19).

### TCID_50_ Endpoint Titration

To determine the tissue culture infectious dose 50 (TCID_50_), 20,000 Vero E6 cells were seeded per 96 wells. 10 µL of SARS-CoV-2 was mixed with 90 µL of PBS or compound and incubated for 30 min at room temperature. Then, the mixture was titrated fivefold, and 18 μL of each dilution was used for inoculation in triplicates in a total volume of 180 µL. Cells were incubated for 6 days and monitored for CPE. TCID_50_/mL was calculated according to Reed and Muench, and detection limits were determined by minimal applied virus dilution or cytotoxicity of the present compound.

### SARS-CoV-2 Infection Assay

To assess infection rate, virus-induced cell death was determined by quantifying cell viability via MTS [3-(4,5-dimethylthiazol-2-yl)-5-(3-carboxymethoxyphenyl)-2-(4-sulfophenyl)-2H-tetrazolium] assay. To this end, 18,000 TMPRSS2-expressing Vero E6 cells were seeded in 96-well plates. The next day, the respective compound of interest was added, and the cells were inoculated with the desired multiplicity of infection (MOI) of SARS-CoV-2 in a total volume of 180 μL. After 2.5 days, when CPE was visible, 36 µL of CellTiter 96 AQueous One Solution Reagent (Promega G3580) was added to the medium and incubated for 3 h at 37°C. Then, optical density (OD) was recorded at 620 nm using an Asys Expert 96 UV microplate reader (Biochrom). All values were corrected for the background signal derived from uninfected cells, and untreated controls were set to 100% infection.

### Cell Viability Assay

Cytotoxicity of the compounds was assessed using a cell viability assay measuring ATP levels in cell lysates with a commercially available kit (CellTiter-Glo, Promega). Experiments were performed corresponding to the respective infection assays in the absence of virus.

### Effect of Products A and B on SARS-CoV-2 Infection of HAECs

Immediately before infection, the apical surface of HAECs was washed three times with 200 µL of PBS to remove accumulated mucus. Next, 50 µL of PBS or product and 50 µL of SARS-CoV-2 (MOI 0.07) were added to the apical surface and incubated for 2 h at 37°C before inoculum was removed and cells were washed three times with PBS. After 1, 2, and 3 days, cells were fixed for 30 min in 4% paraformaldehyde in PBS and permeabilized for 10 min with 0.2% saponin and 10% FCS in PBS (perm/staining buffer). Cells were washed twice with PBS and stained for SARS-CoV-2 spike protein (ab252690, Abcam) diluted 1:300 in staining buffer overnight at 4°C. After two PBS washes, cells were stained with AlexaFluor488-labeled anti-rabbit anti-rat secondary antibody (1:500; Thermo Scientific) and DAPI + phalloidin AF 405 (1:5,000; Thermo Scientific) for 1 h at room temperature. Images were taken on an inverted confocal microscope (Leica TCS SP5) using a ×40 lens (Leica HC PL APO CS2 40x1.25 OIL). Images for the blue (DAPI) and green (AlexaFluor488) channel were taken using appropriate excitation and emission settings that were kept constant for all the acquisitions. For quantification, randomly chosen locations in each filter were selected and z-stacks acquired. A maximum z-projection was performed, and anti-SARS-CoV-2-positive cells per area (0.15 mm^2^) were visually identified and counted.

## RESULTS

**Figure 1.**
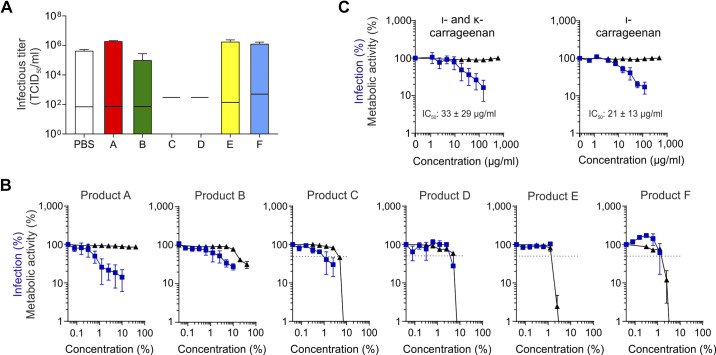
Effect of nasal and oral sprays as well as carrageenans on SARS-CoV-2. *A*: SARS-CoV-2 was incubated for 30 min in 90% PBS or products A–F. The remaining infectious titer was determined by TCID_50_ analysis on Vero E6 cells. Values shown are means ± SD derived from three independent experiments (*n* = 3), each performed in technical triplicates. Black lines indicate detection limits that increase upon cytotoxicity of the respective compound that was observed by light microscopy. *B*, *C*: TMPRSS2-expressing Vero E6 cells were treated with indicated concentrations of products A–F (*B*) or carrageenans (*C*) and infected with SARS-CoV-2. Infection rates were determined 2.5 days later by MTS assay (blue squares). For determination of toxicity, cells were treated with indicated concentrations of compounds in the absence of virus, and cellular ATP was measured by the CellTiter-Glo assay 2.5 days later (black triangles). Values shown in *B* and *C* are means ± SEM derived from two (products C, D, E, and F) or three (products A and B, ι- and κ-carrageenan, and ι-carrageenan) independent experiments (*n* = 2–3), each performed in technical triplicates. TCID_50_, tissue culture infectious dose 50.

To address whether commercially available, topically applied pharmaceuticals affect SARS-CoV-2, we first determined the virucidal activity of five nasal sprays (products A, C–F) and one oral spray (product B) (Table 1). To this end, high titers of the SARS-CoV-2 isolate France/IDF0372 were incubated for 30 min in 90% (v/v) PBS or products A–F. Remaining infectivity was determined by measuring the tissue culture infectious dose 50 (TCID_50_) on Vero E6 cells. Incubation with products A, B, E, and F resulted in similar infectious titers as incubation in PBS, showing that these sprays have no direct virucidal activity (Fig. 1*A*). Products C and D inactivated SARS-CoV-2 infectivity entirely; however, they also affected cell viability (observed by light microscopy), so that the detection limit increased to 2 × 10^3^ TCID_50_/mL (Fig. 1*A*, black lines), corresponding to a reduction of the viral titer by at least 99.5%.

We next explored whether the sprays may inhibit SARS-CoV-2 infection. For this, the products were titrated on TMPRSS2-expressing Vero E6 cells that were subsequently infected with SARS-CoV-2. Viral infection was determined 2.5 days later by MTS assay (20). Simultaneously, cell viability in the presence of the products but absence of virus was determined by quantifying intracellular ATP concentrations. Final cell culture concentrations of products D–F that exceeded ∼2%–5% (v/v) resulted in massive cell death precluding any reliable conclusion regarding antiviral activity (Fig. 1*B*). Product C, which was virucidal (Fig. 1*A*), was also cytotoxic (half-maximal cytotoxic concentration, CC_50_ ∼ 4.4 ± 0.15%) but reduced viral infection with a half-maximal inhibitory concentration (IC_50_) value of 1.3 ± 0.7%, corresponding to a selectivity index (SI) of 3.3. The nonvirucidal products A (a nasal spray) and B (a mouth spray) inhibited SARS-CoV-2 infection with IC_50_ values of ∼1.3 ± 0.8% (v/v; corresponding to a ∼77-fold dilution of product A) and ∼3.1 ± 1.7% (v/v, corresponding to ∼32-fold dilution of product B), respectively. Product A did not affect Vero E6 cell viability at concentrations up to 50% (2-fold dilution), whereas product B reduced cell viability with a CC_50_ value of ∼ 19.3% (∼5-fold dilution, SI ∼6.2).

Products A and B contain carrageenans (Table 1), which are sulfated polysaccharides isolated from red seaweeds previously shown to exert antiviral activity (14, 21–25). Product A contains ι-carrageenan (1.2 mg/mL) and κ-carrageenan (0.4 mg/mL), and product B contains ι-carrageenan only (1.2 mg/mL). To evaluate whether these polyanions exert antiviral activity against SARS-CoV-2, we analyzed purified ι- and κ-carrageenan as well as ι-carrageenan only, without the additives of the products (Fig. 1*C*). Both carrageenan solutions reduced SARS-CoV-2 infection with IC_50_ values of 21 ± 13 µg/mL and 33 ± 28 µg/mL and did not affect cell viability at concentrations up to 160 µg/mL and 120 µg/mL, respectively (Fig. 1*C*). The antiviral activities of both carrageenan preparations are similar to those of products A and B with calculated IC_50_ values of 20 ± 13 µg/mL and 37 ± 20 µg/mL, respectively. Thus, ι- and κ-carrageenans inhibit SARS-CoV-2 infection and are responsible for the antiviral activity in products A and B.

**Figure 2.**
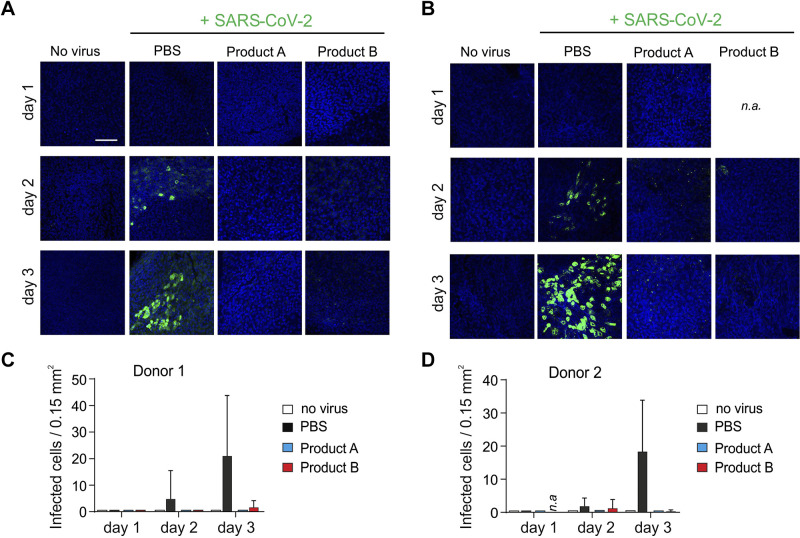
Products A and B inhibit SARS-CoV-2 infection of primary human airway epithelial cultures (HAECs). *A* and *B*: HAECs derived from *donor 1* (*A*) and *donor 2* (*B*) were exposed to PBS or 50% (v/v) of product A or B and then infected with SARS-CoV-2. After 2 h, virus and compound mixture were removed, and cells were washed in PBS to restore the air-liquid interface. After 1, 2, and 3 days, filters were fixed and stained for SARS-CoV-2 spike protein (green) and cell nuclei (blue) and imaged by confocal microscopy (*n* = 1 per donor). Shown are merged images. Scale bars represent 100 µm. n.a., not available. *C* and *D*: number of infected cells per area was obtained by counting SARS-CoV-2-infected cells within microscopic images. Data represent analysis of 3–5 images per timepoint and condition and are means ± SD.

We next tested whether products A and B may also prevent SARS-CoV-2 infection of physiologically relevant target cells. For this, we generated from two donors, fully differentiated human airway epithelial cultures (HAECs) that morphologically and functionally resemble the entry site for SARS-CoV-2 (21, 26). Cultures were exposed at the air-liquid interface to either PBS or a twofold dilution [50% (v/v)] of products A or B and were then inoculated with SARS-CoV-2. Then, 1, 2, and 3 days later, cultures were stained for nuclei (DAPI) and SARS-CoV-2 spike protein, as described (27), and then imaged by confocal microscopy (Fig. 2). At *day 2*, infected HAECs from both donors stained clearly positive for viral spike protein when treated with PBS. The signal intensities (Fig. 2, *A* and *B*) and the number of infected cells (Fig. 2, *C* and *D*) further increased at *day 3*, demonstrating productive infection. Products A and B blocked SARS-CoV-2 infection entirely (Fig. 2, *A* and *C*) in HAECs from *donor 1*, whereas a few spike-positive cells could be detected in HAECs from *donor 2* (Fig. 2, *B* and *D*). Thus, SARS-CoV-2 infection of fully differentiated airway epithelial cell cultures can be efficiently reduced by carrageenan-containing nasal and mouth sprays.

## DISCUSSION

As SARS-CoV-2 primarily enters the human body via infection of nasal epithelial cells (5, 28), we here evaluated whether nasal sprays may exert antiviral activity against this novel pathogen. We found that carrageenan-containing products A (a nose spray) and B (a mouth spray) inhibit SARS-CoV-2 infection of human airway epithelial cultures, which represent a physiologically relevant entry site for SARS-CoV-2. Both over-the-counter products were applied as twofold dilution at the air-liquid interface of the epithelia, and at these concentrations, both products efficiently blocked SARS-CoV-2 infection of HAECs derived from two donors. The limited availability of these primary epithelia did not allow for testing of further dilutions of the sprays and hence to determine IC_50_ values. However, dose-response inhibition studies performed in a cell line showed that a ∼77-fold dilution of product A suppressed SARS-CoV-2 half-maximally and a 10- to 20-fold dilution suppressed SARS-CoV-2 by more than 80%, suggesting that application of the spray into the nostrils might reach local concentrations on nasal epithelia that are sufficient to block SARS-CoV-2 infection.

Products C and D showed virucidal effects upon incubation of virus in 90% (v/v) of the compounds. This virucidal effect is likely mediated by the ingredients xylometazoline hydrochloride and dexpanthenol (29) (present in product D) or the additive benzalkonium (30) (present in products C and D), all of which have previously been described as virucidal (29, 30). Similar antiviral activities against SARS-CoV-2 were also reported for povidone-iodine-containing sprays (present in product D), probably because of the disinfectant properties (31). Upon application of diluted nose sprays, antiviral activity was lost for product D but not for product C, which showed an IC_50_ value of 1.3 ± 0.7% (v/v). However, both sprays diminished cell viability at concentrations exceeding 5% (v/v) in cell culture, possibly due to the ingredient benzalkonium, a known cytotoxic preservative in both sprays (32–34). Also, the microgel-containing products E and F were cytotoxic under conditions tested, precluding any conclusions regarding a possible anti-SARS-CoV-2 effect. It should be mentioned, however, that the cytotoxic effects of products C–F obtained in our in vitro cell cultures assays do not reflect toxicity in vivo, since all sprays analyzed are tested for safety in humans. Furthermore, we emphasize that a repeated administration of nasal sprays (or respective drops) containing decongestants may have harmful effects on the mucosa, which may inadvertently foster infection (35–37).

Carrageenan-containing products A and B inhibited SARS-CoV-2 infection of Vero E6 cells with IC_50_ values of 1.3 ± 0.8% (corresponding to 20 ± 13 µg/mL of ι-/κ-carrageenan) and 3.1 ± 1.7% (corresponding to 37 ± 20 µg/mL), respectively. The anti-SARS-CoV-2 activity of purified ι-/κ-carrageenans was in the same range, showing that these polymers are the responsible antiviral factors in products A and B. Carrageenans have previously been reported to have broad antiviral activity against, e.g., influenza A, dengue, hepatitis A, and rhino- and common cold coronaviruses in cell culture and some clinical studies (16, 22, 24, 38), and application of carrageenan-containing nose sprays to combat SARS-CoV-2 has been suggested (39–41). Four preprint articles support our findings and show that a mixture of gellan and λ-carrageenan (42) or ι-carrageenan inhibits SARS-CoV-2 infection (25, 43, 44). The antiviral effect of carrageenans is most likely based on decreased viral attachment to and entry into target cells. ι-carrageenan has been shown to interfere with papilloma or rhinovirus binding and entry due to its sulphated polysaccharide characteristics that mimic cellular heparan sulfates or aggregates of viral particles (21, 23). Viral binding by ι-carrageenan has also been shown for influenza A and human coronavirus OC43 (38, 45). Thus, ι- and κ-carrageenans, which only differ in the number and location of sulfate moieties on the hexose scaffolds, potentially inhibit SARS-CoV-2 by a similar mechanism. This is supported by a recent study that confirmed inhibition and suggested SARS-CoV-2 aggregation by ι-carrageenan (46).

Carrageenan-containing products A and B do not contain potentially harmful decongestants. Furthermore, clinical trials showed that ι-carrageenan-containing sprays have a good safety profile and resulted in symptomatic benefits, reduced duration of symptoms, and reduced viral loads in adult and pediatric patients with common cold symptoms (13–17). Thus, application of product A may be advisable as a prophylactic agent to protect from acquiring SARS-CoV-2, or at the very early stage of viral infection, because it may reduce viral spread and viral loads in the nasal cavity. Notably, development of severe COVID-19 is always associated with viral dissemination from the upper into the lower respiratory tract. Thus, reducing viral infectivity in the nasal cavity by antiviral nasal sprays or in the oral cavity by oral sprays and rinses (47) early in infection may attenuate disease outcome (38), viral spread, or transmission. It has to be considered that sprays applied to the nasal or oral cavity will not be evenly distributed as a protective film but are instead confined to some areas (48–50). Moreover, the deposited substance will be cleared by mucociliary (11, 49, 51) or salivary clearance (52–54). Thus, the protective effect might be temporally restricted and will not replace the effect of wearing a protective mask. Nonetheless, while providing only some protection, application of the sprays on already infected areas might prevent local spread of the virus, potentially reducing viral loads and thus symptoms or transmission to another individuum.

In conclusion, ι-/κ-carrageenan-containing sprays might be useful repurposed pharmaceuticals for prevention and treatment of SARS-CoV-2/COVID-19, and animal and clinical studies are urgently required to evaluate efficacy in both settings. Finally, it should also be considered to improve the current formulations by combination of carrageenans with other anti-SARS-CoV-2 agents, e.g. gelating agents (42), molecular tweezers (55), peptides (26, 56), or neutralizing antibodies (8, 57).

## DATA AVAILABILITY

All data are available upon request to the qualified researcher.

## GRANTS

This project has received funding through a Collaborative Research Centre Grant of the German Research Foundation (316249678 – SFB 1279) and from the European Union’s Horizon 2020 research and innovation programme under Grant Agreement No. 101003555 (Fight-nCoV) to J.M. and A.N.Z. J.A.M. is indebted to the Baden-Württemberg Stiftung for the financial support of this research project by the Elite Program for Postdocs. D.S., C.C., T.W., L.W., and R.G. are a part of and R.G. is funded by a scholarship from the International Graduate School in Molecular Medicine Ulm. J.M. and M.F. further acknowledge funding by the Ministry for Science, Research and the Arts of Baden-Württemberg, Germany, and the German Research Foundation (458685747 – Fokus-Förderung COVID-19).

## DISCLOSURES

No conflicts of interest, financial or otherwise, are declared by the authors.

## AUTHOR CONTRIBUTIONS

D.S., C.C., R.G., S.S., A.Z., J.A.M., and J.M. conceived and designed research; D.S., C.C., G.F., R.G., T.W., L.W., S.S., A.Z., T.K.H., and J.A.M. performed experiments; D.S., C.C., G.F., R.G., T.W., L.W., S.S., A.Z., T.K.H., and J.A.M. analyzed data; D.S., C.C., G.F., R.G., T.W., L.W., S.S., A.Z., T.K.H., and J.A.M. interpreted results of experiments; D.S., C.C., T.W., and J.A.M. prepared figures; D.S., C.C., S.S., M.F., J.A.M., and J.M. drafted manuscript; D.S., C.C., G.F., R.G., T.W., L.W., S.S., A.Z., T.K.H., M.F., J.A.M., and J.M. edited and revised manuscript; D.S., C.C., G.F., R.G., T.W., L.W., S.S., A.Z., T.K.H., M.F., J.A.M., and J.M. approved final version of manuscript.
